# Morphological features of the photoplethysmographic signal: a new approach to characterize the microcirculatory response to photobiomodulation

**DOI:** 10.3389/fphys.2023.1175470

**Published:** 2023-09-25

**Authors:** Zehava Ovadia-Blechman, Yermiyahu Hauptman, Neta Rabin, Gal Wiezman, Oshrit Hoffer, S. David Gertz, Benjamin Gavish, Lilach Gavish

**Affiliations:** ^1^ School of Medical Engineering, Afeka Tel‐Aviv Academic College of Engineering, Tel Aviv, Israel; ^2^ ACLP—The Center for Language Processing, Afeka Tel‐Aviv Academic College of Engineering, Tel Aviv, Israel; ^3^ Department of Industrial Engineering, The Iby and Aladar Fleischman Faculty of Engineering, Tel‐Aviv University, Tel Aviv, Israel; ^4^ School of Electrical Engineering, Afeka Tel‐Aviv Academic College of Engineering, Tel Aviv, Israel; ^5^ Faculty of Medicine, Institute for Research in Military Medicine (IRMM), The Hebrew University of Jerusalem and the Israel Defense Forces Medical Corps, Jerusalem, Israel; ^6^ The Saul and Joyce Brandman Hub for Cardiovascular Research and the Department of Medical Neurobiology, Faculty of Medicine, Institute for Medical Research (IMRIC), The Hebrew University of Jerusalem, Jerusalem, Israel; ^7^ Yazmonit Ltd, Jerusalem, Israel

**Keywords:** photoplethysmography, waveform, photobiomodulation, low-level laser, entropy, signal processing, peripheral microcirculation

## Abstract

**Introduction and Objectives:** Advanced analysis of the morphological features of the photoplethysmographic (PPG) waveform may provide greater understanding of mechanisms of action of photobiomodulation (PBM). Photobiomodulation is a non-ionizing, red to near-infrared irradiation shown to induce peripheral vasodilatation, promote wound healing, and reduce pain. Using laser Doppler flowmetry combined with thermal imaging we found previously in a clinical study that PBM stimulates microcirculatory blood flow and that baseline palm skin temperature determines, at least in part, why some individuals respond favorably to PBM while others do not. “Responders” (n = 12) had a skin temperature range of 33°C–37.5°C, while “non-responders” (n = 8) had “cold” or “hot” skin temperature (<33°C or >37.5°C respectively). The continuous PPG signals recorded from the index fingers of both hands in the original clinical study were subjected to advanced post-acquisitional analysis in the current study, aiming to identify morphological features that may improve the accuracy of discrimination between potential responders and non-responders to PBM.

**Methods:** The PPG signals were detrended by subtracting the lower envelope from the raw signal. The Root Mean Square (RMS) and Entropy features were extracted as were two additional morphological features -- Smoothness and number of local extrema per PPG beat (#Extrema). These describe the signal jaggedness and were developed specifically for this study. The Wilcoxon test was used for paired comparisons. Correlations were determined by the Spearman correlation test (r_s_).

**Results:** The PPG waveforms of responders to PBM had increased amplitude and decreased jaggedness (Baseline vs. 10’ post-irradiation: Entropy, 5.0 ± 1.3 vs. 3.9 ± 1.1, *p* = 0.012; #Extrema, 4.0 ± 1.1 vs. 3.0 ± 1.6, *p* = 0.009; RMS, 1.6 ± 0.9 vs. 2.3 ± 1.2, *p* = 0.004; Smoothness, 0.10 ± 0.05 vs. 0.19 ± 0.16, *p* = 0.016). In addition, unilateral irradiation resulted in a bilateral response, although the response of the contralateral, non-irradiated hand was shorter in duration and lower in magnitude. Although subjects with ‘cold,’ or ‘hot,’ baseline skin temperature appeared to have morphologically distinct PPG waveforms, representing vasoconstriction and vasodilatation, these were not affected by PBM irradiation.

**Conclusion:** This pilot study indicates that post-acquisitional analysis of morphological features of the PPG waveform provides new measures for the exploration of microcirculation responsiveness to PBM.

## 1 Introduction

Photoplethysmography (PPG) is a non-invasive, inexpensive optical technique that can detect relative changes in the quantity of red blood cells (RBC) in the peripheral microcirculation (Allen, 2007; Nitzan and Ovadia-Blechman, 2022). PPG is widely accepted for monitoring pulsations associated with local blood volume changes assuming a constant RBC concentration (Ovadia et al., 1995; Kyriacou and Chatterjee, 2022). Previous studies have shown that PPG can detect compensatory changes in local blood flow as well as predict changes in systemic hemodynamic variables during a variety of physiological and pathological conditions (Ovadia et al., 1995; Allen, 2007; Ovadia-Blechman et al., 2015a; Ovadia-Blechman et al., 2015b; Ovadia-Blechman et al., 2017; Ovadia-Blechman et al., 2018; Ahmed et al., 2022). Moreover, using PPG monitoring, changes in peripheral blood supply following a unilateral clinical intervention can exert significant contralateral effects, the characterization of which may be further elucidated by advanced post-acquisitional analysis of the PPG signals (Ahmed et al., 2022; Allen, 2022; Mejia-Mejia et al., 2022).

In recent years, advanced morphological analysis of the PPG signal has contributed to a better understanding of the underlying physiological mechanisms associated with various normal and pathological conditions with particular effort seen in various fields of cardiovascular medicine (Hickey et al., 2015; Goshvarpour and Goshvarpour, 2020; Qawqzeh et al., 2020; Vasyltsov and Lee, 2020; Ahmed et al., 2022; Allen, 2022; Mejia-Mejia et al., 2022; Roy et al., 2022). Alterations in features such as entropy, that reflects the level of signal disorder, allow for more precise characterization of the morphology of the signals that may improve clinical diagnosis (Wei et al., 2020; Hauptman et al., 2019).

Photobiomodulation (PBM) is a non-ionizing, red to near-infrared optical irradiation that stimulates and/or stabilizes mitochondrial membrane potential and ATP production, reduces pro-inflammatory mediators, and increases cell proliferation (Pastore et al., 1996; Gavish et al., 2004; Gavish et al., 2008; Passarella and Karu, 2014; Hamblin, 2018; Gavish et al., 2021). It is widely used clinically to reduce pain and accelerate wound healing (Avci et al., 2013; Chow, 2016; Oyebode et al., 2021). PBM was shown to stimulate vasodilatation and increase peripheral blood flow (Schindl et al., 1998; Schindl et al., 2002; Samoilova et al., 2008a; Samoilova et al., 2008b). This effect is based at least in part on the upregulation by PBM of synthesis and secretion of nitric oxide (NO) and modification of reactive oxygen species (Vladimirov et al., 2000; Chen et al., 2008; Gavish et al., 2008; Chen et al., 2022; Keszler et al., 2022). In a previous study using laser Doppler flowmetry and thermal imaging, PBM was found to increase microvascular flow (Gavish et al., 2020) and that those that did not respond were found to have either ‘hot’ or ‘cold’ hand with skin temperature at baseline being >37.5°C or <33°C respectively (Gavish et al., 2020).

The current pilot study involves advanced post-acquisitional analysis of PPG signal recordings collected from the index fingers of both hands in the previous study (Gavish et al., 2020). This post-acquisitional study will interrogate previously validated as well as novel morphological features of the PPG signal. Its purpose is to identify morphological features of the PPG signal that may increase the accuracy of prediction of those likely to respond favorably to PBM treatment and those who may not respond and to quantify the bilateral response using these features.

## 2 Methods

### 2.1 Study overview

This study is a subgroup analysis consisting of advanced post-acquisitional processing of PPG signals collected previously (Gavish et al., 2020), NCT03357523). The study analyses and compares PPG recordings of responders and non-responders (NR) to PBM. The criterion for dividing the participants into responders (n = 12) and non-responders (n = 8) in the original study was an increase of ≥0.5°C which is accepted as a deviation from the normal symmetrical thermal distribution between sides of the body extremities (Ammer, 2012; Garcia Becerra et al., 2022) and was found to indicate underlying pathologies (Suominen and Asko-Seljavaara, 1996; Selfe et al., 2008). In the original study (Gavish et al., 2020), this criterion was used as a threshold for dynamic changes in thermal response to PBM.

This created 3, non-overlapping subgroups according to baseline skin temperature. “Responders” to PBM had a range of skin temperature from 33°C to 37.5°C, while “non-responders” to PBM had “cold” or “hot” skin temperature (<33°C or >37.5°C respectively). The original study was approved by the Afeka Institutional Ethics Review Board (05.04.2017-1-AFK), and all participants signed an informed consent form prior to inclusion.

The study population included 20, healthy, adult, non-smoking volunteers (10:10 males: females, 30 ± 8 years old) that were randomized to receive either red or near infrared PBM irradiation (633 nm, power density = 70 mW/cm^2^; total energy per session 21 J/cm^2^; 830 nm, 55 mW/cm^2^; 16.5 J/cm^2^) using a commercial light emitting diode (LED) cluster (Omnilux new-U, Photomedex United States). The LaserMate power-meter (Coherent, Auburn group, Coherent-Europe, Utrecht, Holand) was used to confirm the power density at the plane of irradiation.

Subjects were requested not to consume any beverages that contained caffeine (coffee, tea, cola, etc.) or alcohol at least 3 h prior to the session. The subjects sat relaxed in a quiet room with constant temperature (25°C ± 1°C) for at least 15 min with exposed hands before data collection. The LED cluster was positioned over the left wrist and switched on for 5 min, while the right hand was protected from the light.

The PPG optical reflective sensors (model SS4LA, wavelength—860 nm) were placed on the index finger of both hands using Velcro® strip (Biopac™ System Inc., Goleta, CA). Both PPG and electrocardiographic (ECG) signals were monitored continuously and sampled at 200 Hz using the Biopac™ software. Skin temperature was measured in the center of the palm by an infrared thermal imaging camera (emissivity = 0.98, Sensitivity = 50 mK) that was positioned above the hands (FLIR A35, FLIR Systems Inc., OR, United States). The thermal camera calibration has undergone factory calibration to be within the manufacturer’s accuracy. Signals were collected before PBM irradiation and during irradiation and continued for 20 min after the end of irradiation. The signal processing was performed at the following time periods: half a minute before the irradiation (baseline), the last 3 min of the irradiation, and at follow-up–during 5–10 min, 10–15 min, and 15–20 min after the end of irradiation.

### 2.2 Signal processing and feature extraction

**FIGURE 1:**
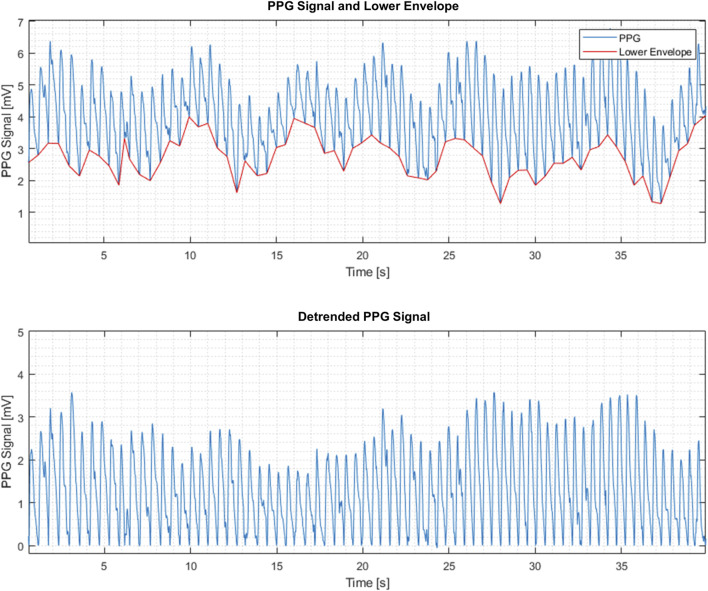
Raw (top) and detrended (bottom) PPG signals.

In general, PPG signals have beats reflecting local blood volume variations in response to the blood pressure fluctuations during the cardiac cycle. PPG maximum and minimum reflect the systolic and diastolic pressures respectively (Figure 1). The PPG beats are superimposed on slower signal variations generated by other dynamic processes, e.g., respiratory activity (“lower envelope” in Figure 1). In addition, the PPG signal contains local minima and maxima that may be physiologically meaningful and that can be studied by the presently described extraction of PPG-signal features.

The PPG signal processing was performed as follows:

a. Sampling the PPG signal at 200 Hz and dividing it into consecutive frames. The frame size was 120 s with 3 s stride (97.5% overlap). Thus, each frame consists of N = 24000 samples.
b. Restricting the analysis to the PPG beats by subtracting the lower envelope from the raw PPG signal resulted in a detrended PPG signal, denoted by x(n), where n = 1,2, … N as the sample number (Figure 1). The lower envelope is constructed by finding the lowest point in each PPG beat and connecting these points consecutively by a piecewise linear function.
c. Extracting the following features from each frame of detrended PPG signals:

#### 2.2.1 RMS

RMS stands for the Root Mean Square of a frame of N samples and calculated by Eq. 1 from the detrended PPG signal.

$$RMS=\sqrt{\frac{1}{N}\sum_{n=1}^{N}x^{2}\left(n\right)}$$ (1)

#### 2.2.2 Entropy

Entropy is a statistical measure of the randomness of data. This feature indicates the extent of disorder of the signal. Entropy is calculated by Eq. 2, where the Probability Mass Function (PMF), p(i), is the normalized histogram (using 256 bins—i.e., dividing the signal level range into 256 equal parts) of the detrended PPG signal *x(n)*.

$$Entropy=-\sum_{i=1}^{256}p\left(i\right)*\log_{2}\left(p\left(i\right)\right)$$ (2)

#### 2.2.3 Smoothness

The average of High Frequency Components (HFC) of the signal is obtained by averaging the absolute values of the differences between the detrended PPG signal $x\left(n\right)$ and a filtered signal $x^{\prime }\left(n\right)$ given by Eq. 3.

$$Smoothness=\frac{1}{N}\sum_{n=1}^{N}\left|x\left(n\right)-x^{\prime }\left(n\right)\right|$$ (3)

A moving-average filter given by Eq. 4 is used, where the value of k was set to 30, and x is the detrended PPG signal.

$$x^{\prime }\left(n\right)=\frac{1}{2k+1}\sum_{l=-k}^{+k}x\left(n+l\right)$$ (4)

#### 2.2.4 Local extrema

This feature counts the number (#) of Extrema points (local maxima or minima) in each beat. The calculation was performed as follows:

(1) The derivative of the detrended PPG signal was calculated using the local polynomial method (De Brabanter et al., 2013).
(2) The sign of the derivative of the detrended PPG signal was calculated. Positive values were set to 1 and negative values were set to −1.
(3) The time index of the sign changes (i.e., from −1 to 1 or *vice versa*) was marked as an extremal point.
(4) If two Extrema points were detected in a time interval that was smaller than 50 ms, only one point was accounted.
(5) The number of Extrema was counted in the calculation frame.
(6) The number of Extrema was divided by the mean heart-rate to obtain the average number per PPG beat.

(See Discussion for additional points related to the morphological features defined in this section.)

### 2.3 Study outcomes

Study outcomes included the average feature values and the average change from baseline during irradiation and 10, 15, and 20 min during the follow up period.

### 2.4 Statistics

All participants of the original trial were included. Variables are presented as mean ± SD. Statistical analysis pertained only to the “responders” in view of the sample size. Normality of the data was determined with the Shapiro-Wilk test with a cutoff of *p* = 0.1. The Friedman’s test with multiple comparisons was used to compare baseline to other time points (and Holm’s modification of Bonferroni’s correction for 4 comparisons [HMBC]) and the exact Wilcoxon signed rank test was used to compare irradiated to non-irradiated hands (ratio to baseline) per time point also with HMBC. The Spearman correlation test was used to determine the level of correlation between hands. In order to determine if the improvement in correlation between pre- and post-irradiation was significant, Spearman correlation coefficients (*r*_s_) were computed twice between the right and left hands for each PPG feature—once for pre-irradiation and once for post-irradiation. These were compared using a special adaptation of Fisher’s *z*-transformation (Dunn and Clark, 1969) that can be assumed to be normally distributed in such cases (Silver et al., 2004) and which is suitable for Spearman coefficients (Zar, 2010; Wilcox, 2016) (See supplement). *p*< 0.05 was considered significant.

## 3 Results

**FIGURE 2:**
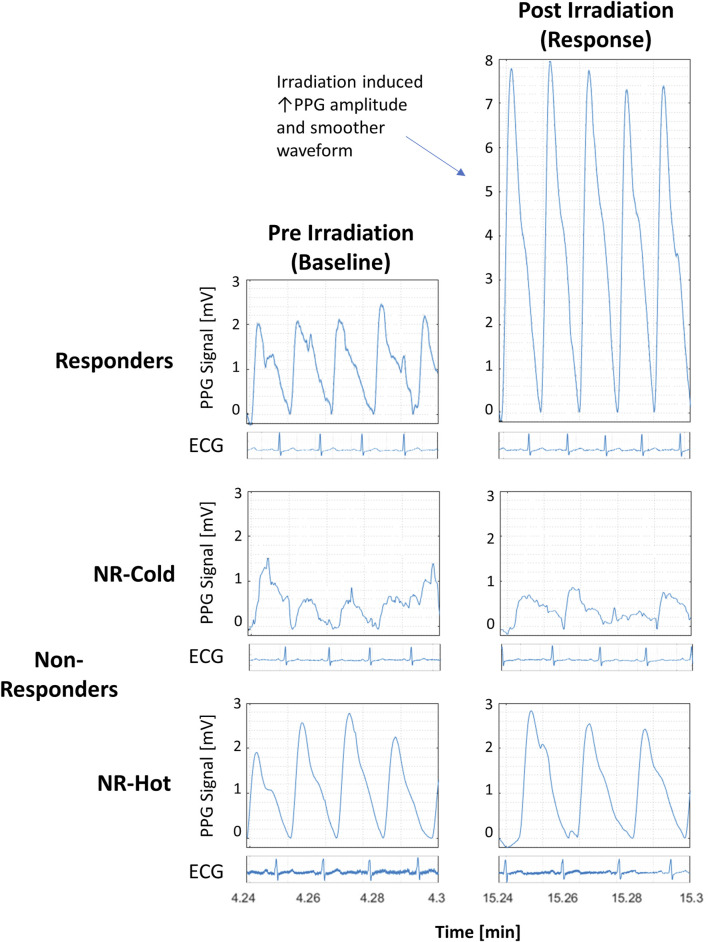
Detrended PPG signals from responders and non-responders (NR) to photobiomodulation (PBM) before and 10 min after PBM from 3 participants with accompanying electrocardiographic strips to show synchronicity with the pulse. All graphs are at the same scale.

**FIGURE 3:**
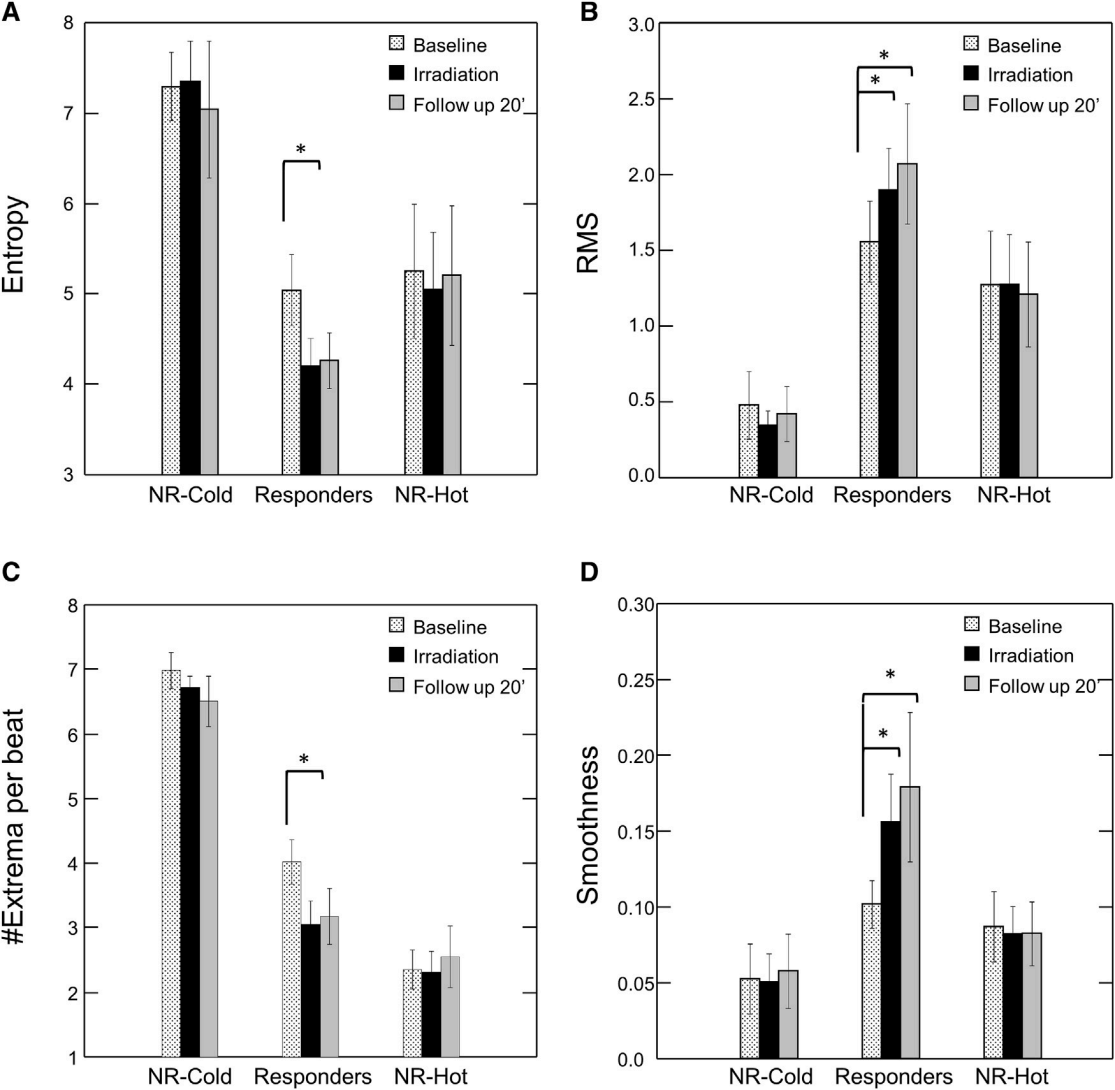
Effect of Photobiomodulation on Morphological Features of the PPG Waveform by Subgroup. Bars represent mean ± SEM for each subgroup at baseline (dots), after irradiation (black), and at the end of the follow up period (20’ post-irradiation) (grey) for **(A)** Entropy; **(B)** Root Mean Square (RMS); **(C)** #Extrema per beat; and **(D)** Smoothness. Note significant change in each of the features post-irradiation for the “responders”. *p < 0.05 by Friedman’s test with multiple comparisons and Holm’s modification of Bonferroni’s correction. NR = non-responders.

Representative examples of the distinctive PPG waveform for each group before and after irradiation are depicted in Figure 2, and the extracted morphological features, including Entropy, Root Mean Square (RMS), Smoothness, and the number of Extrema per beat (#Extrema) for each time period (baseline, irradiation, and follow up), are presented in Table 1 and Figures 3A–D).

**TABLE 1:** Effect of photobiomodulation on the morphological features of the photoplethysmographic waveform.

PPG feature	Irradiated (PBM)				Non-irradiated				Correlation r_s_	
	Pre	Post	%	p*	Pre	Post	%	p*	Pre	Post
Entropy	5.0 ± 1.3	3.9 ± 1.3	−21%	0.012	5.6 ± 1.6	4.9 ± 1.5	−11%	0.008	0.706	0.853
#Extrema	4.0 ± 1.1	3.0 ± 1.6	−27%	0.009	3.6 ± 1.36	2.87 ± 1.34	−19%	<0.001	0.566	0.923†
RMS	1.57 ± 0.89	2.25 ± 1.22	+80%	0.00	0.97 ± 0.5	1.26 ± 0.74	+31%	<0.001	0.469	0.811
Smoothness	0.10 ± 0.05	0.19 ± 0.16	+107%	0.016	0.07 ± 0.04	0.11 ± 0.06	+61%	<0.001	0.860	0.797

PBM = photobiomodulation (low level laser irradiation), Pre = baseline; Post = 10 min after irradiation; % = Change over baseline; RMS = root mean square; Data = mean ± SD; by Spearman correlation test; *p < 0.05 by Friedman’s test with multiple comparisons with Holm’s modification of Bonferroni’s correction; †by modified Fisher’s z-transformation.

Following irradiation, responders (n = 12) had increased amplitude and decreased jaggedness displaying an increase in RMS and smoothness of 80% and 107% respectively. They had a decrease in Entropy and #Extrema of 21% and 27% respectively (Baseline vs- 10’ post-irradiation: Entropy, 5.0 ± 1.3 vs. 3.9 ± 1.1, *p* = 0.012; #Extrema, 4.0 ± 1.1 vs. 3.0 ± 1.6, *p* = 0.009; RMS, 1.6 ± 0.9 vs. 2.3 ± 1.2, *p* = 0.004; Smoothness, 0.10 ± 0.05 vs. 0.19 ± 0.16, *p* = 0.016) (Table 1).

Non-responders (n = 8) with “cold,” or “hot,” baseline skin temperature appeared to have PPG waveforms that were morphologically distinct from responders. Those with “cold” hands (n = 3) appeared to have the highest Entropy and #Extrema, and the lowest RMS and Smoothness, reflecting a noisy and low-amplitude PPG waveform. By contrast, the signal of those with hot’ hands (n = 5) appeared noiseless with the lowest number of Extrema. However, the numbers of subjects in each of these two extreme groups (on the high versus the low end of baseline skin temperature) were too small to reach definite conclusions regarding the differences between these two groups of non-responders. Nonetheless, it should be noted that the morphological features of the PPG in these two extreme groups of non-responders were not affected by PBM irradiation.

### 3.1 Bilateral effect

**FIGURE 4:**
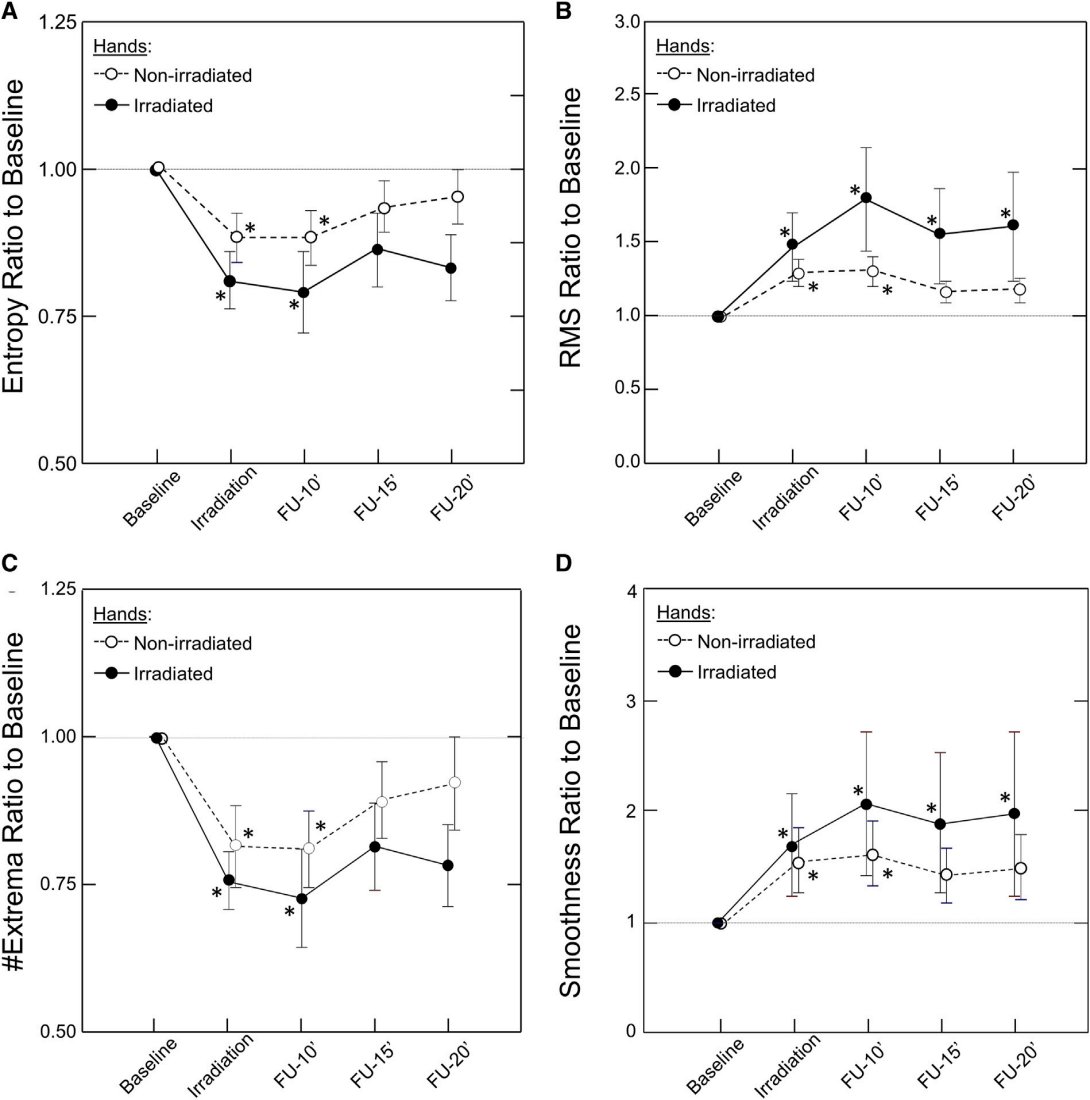
Kinetics of the Bilateral Response of Morphological Features of the PPG Signal to Unilateral Photobiomodulation. Note the significant change post irradiation for both hands that remained significant throughout the experiment in the irradiated hand but only up to 10 min in the non-irradiated hand. Also note that the response was larger in the irradiated than the non-irradiated hand. Data points and error bars represent mean ± SEM by time point for the irradiated hands (closed circles, solid line) and non-irradiated hands (open circles, dashed line) of responders to PBM (n = 12) for **(A)** Entropy; **(B)** Root Mean Square (RMS); **(C)** #Extrema per beat; and **(D)** Smoothness. *p < 0.05 by Friedman’s test with multiple comparisons and Holm’s modification of Bonferroni’s correction.

Unilateral irradiation resulted in a bilateral response, but the response of the contralateral, non-irradiated hand was shorter in duration, persisting for only 10 min, and lower in magnitude (Figure 4). The correlation between irradiated and non-irradiated hands in Entropy, #Extrema, and RMS was moderate before irradiation and strong post-irradiation (Table 1). This was not the case with Smoothness since the correlation was already strong at baseline (r_s_ = 0.86). The strongest improvement of correlation after irradiation between irradiated and contralateral, non-irradiated hands was seen in the #Extrema feature (r_s,_ baseline vs. post-irradiation: 0.566 vs. 0.923, *p* = 0.018).

## 4 Discussion

In recent years, photoplethysmography (PPG) has become very widely used for non-invasive monitoring instruments particularly in the healthcare arena. This has included its use as a basis for a rapidly expanding variety of wearable devices (Singh et al., 2021; Charlton and Vaidotas, 2022). The implementation of advanced methods of PPG signal processing, including advanced morphological analysis, has expanded the capabilities, accuracy, and usefulness of this technology in various fields of physiology and pathology such as cardiovascular function, sleep studies, pregnancy, pain, and mental health (Hickey et al., 2015; Qawqzeh et al., 2020; Ahmed et al., 2022; Allen, 2022; Allen and Fei, 2022; Mejia-Mejia et al., 2022).

Previously we found, using laser Doppler and thermal imaging, that PBM induces arteriolar vasodilatation resulting in both immediate and long-lasting increased capillary flow and tissue perfusion in healthy individuals, but not in participants having “cold hands” or “hot hands” (Gavish et al., 2020).

It is important to emphasize that the overall microvascular response to PBM is large in magnitude which is why it can be easily detected even by measurements of skin temperature that are a low-resolution proxy for microvascular blood flow. However, other characteristics of the microvascular response to PBM, for example, change over time or differences between the irradiated limb vs. the contralateral, non-irradiated limb, are much subtler and smaller in magnitude and appear to have a more complex behavior. Hence, an additional direct and more refined method was required.

In the current study, post-acquisitional analysis of advanced (known and novel) morphological features of the PPG waveforms obtained from these three groups was performed to better understand and provide clinically valuable physiological interpretation for the response to PBM. These additional morphological features, or combinations thereof, may be used in the future to improve the ability to identify potential responders to PBM.

The responders were found to have increased amplitude of the PPG signal and decreased level of jaggedness in the PPG waveform, i.e., the signal became smoother.

### 4.1 Morphological features of the PPG signal

The purpose for detrending the PPG signal was to preserve relevant physiological information and to remove the low-frequency components that were not relevant for the analysis. This involved lower envelope subtraction that is similar to a high pass filter but has some additional advantages. The high pass frequency determination is not required, there are no filter transients in the signal, and the remaining upper envelope clearly manifests the amplitude swing of the PPG signal, since the lower envelope is constant. No additional noise removal filtering was used in order to preserve the relevant information (“jaggedness”).

Two common features were used: Root Mean Square (RMS) of the signal and Entropy for the signal shape (Wei et al., 2020; Singh et al., 2021; Mejia-Mejia et al., 2022). Two new features were introduced -- Smoothness and number (#) of local Extrema per PPG beat. The latter are based on the development of a dedicated algorithm for this research that provides more accurate quantitative information regarding the level of jaggedness of the PPG signal. The Smoothness level of a signal is commonly determined by calculating the higher frequency portion of the signal spectrum or calculating the energy after high-pass filtration (Wei et al., 2020; Singh et al., 2021; Mejia-Mejia et al., 2022). These methods require the determination of the cutoff frequency; that is, the frequency bands of the noises and the ‘clean’ signal. In applying these methods to the PPG signals, the determination of the optimal cutoff frequency was found to be difficult due to the nature of these signals, which are not always fully periodic, and due to the jaggedness of the envelope. To overcome this problem, the RMS value of the PPG signal was calculated after subtracting from the PPG signal its smoothed version obtained by a simple moving-average filter. Following this subtraction, a smooth PPG signal with a very low RMS value was obtained since the signal and its smooth version are similar.

The calculation of derivatives of the PPG signal is often an essential step in pulse wave analysis. For example, several indices of vascular aging can be extracted from the second derivative of the PPG pulse wave (Takazawa et al., 1998; Mejia-Mejia et al., 2022). In this study the number (#) of local Extrema per beat was calculated as another method for describing the jaggedness of the signal: Local Extrema can be easily identified by analyzing the derivatives of the PPG signal. Thus, higher jaggedness will result in a higher count of local Extrema.

### 4.2 Physiological interpretation of the PPG features

The morphological features of the PPG signal represent characteristics of blood volume changes in a microvascular bed reflecting instantaneous changes in the number of RBCs during the pulsatile blood flow. In the case of constant RBC concentration, the RMS of the signal represents the mean value of the pulsatile component of the blood volume which decreases for smaller diameter and stiffer arteriolar walls (Fusi and Farina, 2020). Thus, in comparison to responders, RMS is expected to be lower in “cold hands” due to vasoconstriction and the elevated wall stiffness associated with increased vascular tone. It should be higher in “hot hands” due to vasodilation.

Entropy represents a measure of fluctuations in the signal structure that reflects its complexity. Thus, Entropy is likely to increase when blood flow is compromised either because of an intrinsic pathology of the vessels themselves or inadequate supply with respect to physiological demand resulting in attempts at compensation. In the case of “cold hands,” PBM seems to improve the blood flow resulting in reduction of signal complexity (Entropy) from its baseline level.

Regarding the number of Extrema per beat, the pulse waveform always includes a maximum point at the systolic peak and, frequently, a local minimum at the diastolic phase. Additional Extrema are likely to represent instabilities in the blood flow and dynamic fluctuations in the distribution of RBCs which tend to aggregate at low flow states and in “cold hands” during the diastolic phase (Lipowsky, 2005; Baskurt and Meiselman, 2007). Thus, increased blood flow, as occurs in “hot hands”, or upon/after irradiation, is expected to reduce the number of Extrema as observed.

### 4.3 Clinical considerations

PPG signal processing led to two, clinically important findings: First, the duration of the response to irradiation in the group of responders lasted for at least 20 min. The second is the synchronized changes in the PPG signal in both hands following irradiation of only one hand. Previous clinical studies conducted with thermography and laser Doppler flowmetry have shown that photostimulation for at least 15 min elicits a response in the non-irradiated side, albeit smaller in magnitude (Schindl et al., 2002; Samoilova et al., 2008a). This bilateral response has importance in clinical practice when the area requiring treatment is not accessible or is too painful to treat. Although the vasodilative response following PBM was shown to depend on local NO synthesis or release (Samoilova et al., 2008b; Keszler et al., 2018; Weihrauch et al., 2021; Keszler et al., 2022), it has been suggested that the bilateral effect may also point to the involvement of neuronal pathways coursing through the central nervous system (Koltzenburg et al., 1999) and/or the systemic release of a humoral mediator. Previous clinical studies using other interventions also observed bilateral effects where local stimulation on one side affected the other side (Koltzenburg et al., 1999; Huang et al., 2007; Ovadia-Blechman et al., 2018; Ovadia-Blechman et al., 2021). Further studies are in order to identify the precise pathway or mediator that can be manipulated, upregulated, or synthesized to simulate, or to improve the efficiency of, the therapeutic effects seen with PBM.

Methods such as time-frequency analysis and machine learning (Allen et al., 2021) should be of additional benefit for enhanced automaticity, standardization of interpretation of PPG morphological data, and more precise prediction of response to PBM.

The main limitation of our study was the small sample size of the “cold” and “hot” groups that prevented appropriate statistical evaluation of the effects of PBM on the morphological features of the PPG signal in these two extreme groups. For this reason, the results of this study were based on the post-acquisitional analysis of the PPG waveform of the responders’ group for which adequate numbers were available and reliable statistical separation obtained.

## 5 Conclusion

This pilot study indicates that post-acquisitional analysis of morphological features of the PPG waveform provides new measures for the exploration of microcirculation responsiveness to PBM. Extraction of morphological features from continuous synchronous bilateral PPG measurements enables additional quantification of the kinetics and magnitude of the local (irradiated hand) and the systemic (non-irradiated) response to intervention. An algorithm for prediction of response to PBM may be developed based on a combination of these features. Application of these advances in waveform analysis to machine learning may improve accuracy, enhance standardization of interpretation, and facilitate automaticity, thereby expanding the usefulness of the rapidly expanding PPG-based non-invasive monitoring technologies for hospital as well as pre-hospital and home care.

## Data Availability

The raw data supporting the conclusion of this article will be made available by the authors, without undue reservation. Requests to access these datasets should be directed to ZOB, zehava@afeka.ac.il
