# Supplementary material for: Morphological features of the photoplethysmographic signal: a new approach to characterize the microcirculatory response to photobiomodulation
Source: Front Physiol. 2023 Sep 25;14:1175470. doi: 10.3389/fphys.2023.1175470 (PMC10561251; doi:10.3389/fphys.2023.1175470)
Supplement: Supplementary file 1 [file DataSheet1.PDF]

Appendix

Comparison between two dependent Spearman correlation coefficients

1. Background
   A. Data
      (a)Sample: 12 patients
      (b)Design: 4 measurements per patient
         Pre: Left hand, Right hand
         Post: Left hand, Right hand
   B. Null Hypothesis: Pre correlation between hands = Post correlation between hands
   C. H_1: Two-tail
2. Procedure
   A. Notation
      (a) Let r_{ab} represent the Spearman correlation coefficients between variable a and variable b

And let	j	correspond to	Pre,	Left hand
	k		Pre,	Right hand
	h		Post,	Left hand
	m		Post,	Right hand

(b) Then	r_{jk}	is the correlation	between Pre-Left and Pre-Right hands
	r_{hm}		between Post-Left and Post-Right hands
	r_{jh}		between Pre-Left and Post-Left hands
	r_{jm}		between Pre-Left and Post-Right hands
	r_{kh}		between Pre-Right and Post-Left hands
	r_{km}		between Pre-Right and Post-Right hands

   B. Computation
      (a) Compute all 6 r_{ab} coefficients
      (b) Define

$$c1 \equiv r_{jh}^2 + r_{jm}^2 + r_{kh}^2 + r_{km}^2$$

$$c2 \equiv r_{jh} r_{km} + r_{jm} r_{kh}$$

$$c3 \equiv r_{jk} r_{jh} r_{jm} + r_{jk} r_{kh} r_{km} + r_{jh} r_{kh} r_{hm} + r_{jm} r_{km} r_{hm}$$

$$c4 \equiv (1 - r_{jk}^2)(1 - r_{hm}^2)$$

      (c) Then

$$c = (0.5 r_{jk} r_{hm} c1 + c2 - c3) / c4$$

   C. Test
      (a) standard deviation

$$\sigma = \sqrt{\frac{2(1.06)(1-c)}{n-3}}$$

         where n = sample size
         Note: expression from Dunn (1969), p368, eq.9
         factor 1.06 from Zar (2010), §19.9, p400
      (b) Fisher's transformation

$$z_{jk} = \tanh^{-1}(\mathrm{r}_{jk})$$

$$z_{hm} = \tanh^{-1}(\mathrm{r}_{hm})$$

         Note: applicable only for $r_{ab} \le 0.9$ and $n \ge 10$
      (c) Finally $z = \frac{z_{jk} - z_{hm}}{\sigma}$ which is N (0,1) under H_0

   D. Results
      (a) p-value
         If $p1$ is the upper tail
         And $p2$ is the lower tail
         Then p=2min($p1$, $p2$)
      (b)Features
         Analyse each of the 4 features separately
